# Non-Additive Entropy Formulas: Motivation and Derivations

**DOI:** 10.3390/e25081203

**Published:** 2023-08-13

**Authors:** Tamás Sándor Biró, Airton Deppman

**Affiliations:** 1 Wigner Research Centre for Physics, 1121 Budapest, Hungary; 2 Institute for Physics, University Babeş-Bolyai, 400294 Cluj, Romania; 3 Instituto de Física, University of São Paolo, São Paolo 05508-090, Brazil; deppman@usp.br

Entropy is a great tool in thermodynamics and statistical physics. Originally conceptualized by Clausius as a state descriptor that was distinguished from heat, it became a basic principle for statistical and informatics calculations. Its classical form, Boltzmann entropy, is widely used. Moreover, its generalizations are numerous: altered, non-logarithmic formulas concerning the relationship between the probability of a given state and the total entropy of a system proliferate.

This Special Issue is devoted to the mathematical background and physical motivation behind using non-additive entropy formulas, which are, in most cases, still group entropies, defined as expectation values, and incorporate, in one way or another, nontrivial correlations in the investigated systems and processes. Despite the vast number of studies on applying such formulas in informatics, mathematics, and statistical physics approaches to a wide range of physical, biological, economical, and social phenomena, we felt that a collection of their derivations, beyond simple axiomatization and usage, would be useful for modern statistical communities.

The concept of entropy has been changing swiftly since C. Tsallis and, before him, a series of mathematicians and informatics experts proposed various generalizations of Boltzmann–Gibbs entropy. The most distinguishing property of these formulas is their non-additivity with respect to factorizing probabilities, with the notable exception of Rényi entropy, which is, on the other hand, not an expectation value.

The determination of a non-additive entropy formula has motivated investigations in many directions. Some works have considered the theoretical and axiomatic implications of the new form of entropy. Others have proposed physical mechanisms that could lead to non-additive entropy. Evidence for the applicability of generalized thermodynamics appears in many branches of physics and other fields, such as biological systems, socio-economic environments, information theory, and complex networks, among many others.

This Special Issue is dedicated to reviewing these developments, sharing new results and opening new perspectives as to the advancement of our knowledge of entropy. We have gathered contributions on the following topics:

(1) The implications and applications of non-additive entropy;
(2) Advances in the theoretical aspects of entropy;
(3) The mathematical aspects of non-additive entropy;
(4) The thermodynamic consequences of generalized entropy;
(5) The origins of non-additivity in the entropy of complex systems.

The contributors to this Special Issue include colleagues who have been working on these ideas and related concepts for decades. The included papers cover issues ranging from derivation discussions of given formula groups to the applications of a given entropy formula for contemporary problems—and solving them—and the statistical data analysis of COVID-19 infections.

We have received eleven papers, including an extended review by Angle R. Plastino and Angelo Plastino titled “on the Connection Between the Microcanonical Ensemble and the $S_{q}$-canonical Probability Distribution” (i.e., the Tsallis–Pareto cut power law distribution). This work is connected to the classical thermodynamics background in the micro-canonical approach, as is the paper by Tamás Sándor Biró entitled “Non-Additive Entropy Composition Rules Connected With Finite Heat-Bath Effects”, which concerns the finite heat capacity of the reservoir determining the Tsallis-*q* parameter. Also of a general background nature, the contribution by Ozgur Afsar and Tirnakli investigates the self-organization process in non-extensive open systems. This paper also reveals the connections to chaotic dynamics and discusses a simple example.

A review-like contribution on the mathematical relations regarding *q*-entropy is presented by Angel R. Plastino, Constantino Tsallis, Rosell S. Wedemann, and Hans J. Hubold, concentrating on entropy optimization issues and the use of generalized logarithms—simultaneously implying generalizationsof the logarithmic entropy formula—and some interesting duality relations between various *q* parameters. In the direction of more abstract mathematical tools, two contributions in this Special Issue cover fractal derivatives. The first of which, which was written by Airton Deppman, Eugenio Megías, and Roman Pasechnik and concerns “Fractal Derivatives, Fractional Derivatives and *q*-Deforemd Calculus”, dives deeper into formalism. A similarly motivated article written by Jin-Wen Kang, Ke-Ming Shen, and Ben-Wei Zhang considers the *h*-Derivative in relation to non-additive entropy. Finally, we have the paper by F. Nobre and E. Curado, wherein the dynamical evolution of non-additive entropies is considered on the basis of a generalized H-theorem, resulting in non-linear equations for a class of systems.

The rest of the submissions deal with applications, but what incredible applications they are! Jin Yan and Christian Beck discuss an information shift model, which they demonstrate to be equivalent with q=3 entropy in a compact phase space. It is intended to be applied to a pre-universe before ordinary spacetime was created and the natural laws as we know them were formed. The authors show that the electromagnetic fine structure constant’s value, 1/137, can be interpreted as a special “equilibrium” state for Chebisev maps describing a chaotic pre-universe. This is quite an interesting perspective.

More earthbound but also more controllable applications are provided in relation to well-studied phenomena. Grzgeorz Wilk and Zbigbiew Wlodarczyk present a review of applying non-extensive entropy to multi-particle production in high-energy collisions inside accelerators. In their contribution, Elif Tuna, Alif Evren, Erhan Ustaoglu, Busra Sahin, and Zehra Zeynep Sahinbasoglu discuss an application with importance in hypothesis testing. Lastly, we have collected a genuine non-physics application to the statistics of COVID-19 infections written by Markos N. Xenakis estimating the risk of indoor infection.

In closing this Editorial, we thank the contributing authors for being responsive to our call and providing such a remarkable variety of thoughtful and interesting papers on non-additive entropy. The invaluable help provided by the MDPI Editorial Office is also gratefully acknowledged.

